# Deciphering the binding mechanism of tafamidis to calf thymus DNA: multimodal spectroscopic, thermodynamic, and computational perspectives

**DOI:** 10.1039/d5ra04723d

**Published:** 2025-08-18

**Authors:** Manal A. Alossaimi, Heba Elmansi, Fathalla Belal, Galal Magdy

**Affiliations:** a Pharmaceutical Chemistry Department, College of Pharmacy, Prince Sattam bin Abdulaziz University Al-Kharj 11942 Saudi Arabia m.alossaimi@psau.edu.sa; b Pharmaceutical Analytical Chemistry Department, Faculty of Pharmacy, Mansoura University Mansoura 35516 Egypt; c Pharmaceutical Analytical Chemistry Department, Faculty of Pharmacy, Kafrelsheikh University Kafrelsheikh 33511 Egypt galal_magdy@pharm.kfs.edu.eg; d Department of Pharmaceutical Analytical Chemistry, Faculty of Pharmacy, Mansoura National University Gamasa 7731168 Egypt

## Abstract

The interaction between small molecules and biological macromolecules is a crucial area of research with significant implications across various scientific disciplines. Tafamidis is a clinically approved drug for transthyretin-mediated amyloidosis, yet its molecular interactions with biological macromolecules such as DNA remain unexplored. Investigating such interactions is crucial for understanding its broader pharmacodynamic profile and potential off-target effects. In this study, the binding interaction between tafamidis and calf thymus DNA (ct-DNA) was investigated, for the first time, using a combination of spectroscopic techniques, viscosity measurements, ionic strength studies, thermodynamic analysis, and computational modeling. The key binding parameters, including the binding constant, number of binding sites, binding forces, and binding mode were determined. UV-vis spectroscopic analysis revealed a binding constant on the order of 10^5^ M^−1^, indicating a moderate-to-strong binding affinity between tafamidis and ct-DNA. Thermodynamic parameters (Δ*H*° > 0 and Δ*S*° > 0) suggested that hydrophobic interactions primarily drive the binding process. Fluorescence spectroscopy, viscosity measurements, and molecular modeling indicated that tafamidis preferentially binds to AT-rich regions of ct-DNA and acts as a minor groove binder. Ethidium bromide displacement assays showed no significant effect of tafamidis on the ethidium bromide–DNA complex, further supporting the groove binding mechanism. Molecular dynamics simulations corroborated these findings. Moreover, molecular dynamics simulations validated the dynamic behavior and stability of the tafamidis–ct-DNA complex, demonstrating its excellent structural stability. Overall, these results enhance the understanding of tafamidis by providing valuable insights into its pharmacological mechanisms and molecular interactions.

## Abstract 2

This work introduces the first study on the binding interaction between tafamidis and calf thymus DNA using multi-spectroscopic, viscosity, ionic strength, thermodynamic, molecular docking, and molecular dynamic simulation approaches.

## Graphical abstract

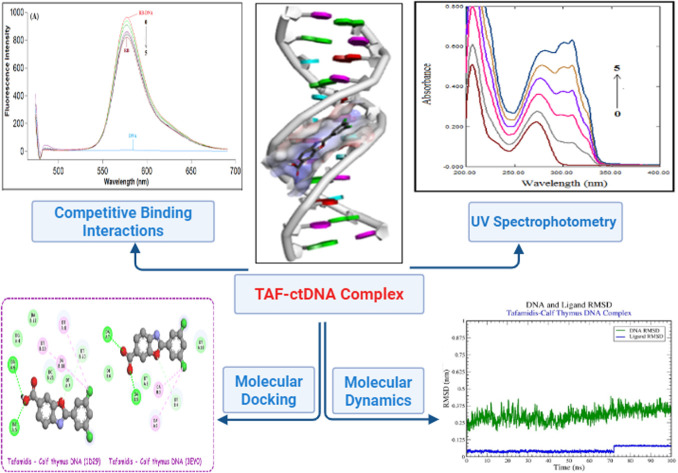

## 1. Introduction

**Fig. 1:**
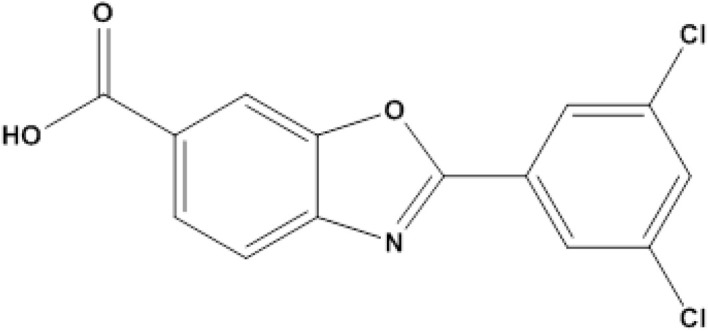
Chemical structure of tafamidis.

Tafamidis, (TAF, Fig. 1) is a medication used to treat transthyretin-mediated amyloidosis, which is a diverse collection of illnesses that result in end-organ damage due to the extracellular accumulation of insoluble misfolded proteins.^1^ It is chemically named as 2-(3,5-dichlorophenyl)-benzoxazole-6-carboxylic acid. It is a non-NSAID small molecule that is orally bioavailable and administered once daily.^2^ This benzoxazole-based small molecule belongs to transthyretin stabilizers. It acts by the prevention of the formation of the transthyretin protein which deposits in the heart. The liver creates a protein called transthyretin (TTR). This protein normally exists as a stable group of four units. However, changes in its shape can cause it to become unstable and break down into individual units. This can happen naturally as we age (called ATTRwt) or due to inherited genetic problems (called ATTRv). It is sold under the trade names: Vyndamax® and Vyndaqel®.^3^

Deoxyribonucleic acid is essential to life because it contains information about ancestry. One of the main areas of research in the last few decades has been the process by which certain tiny molecules bind to DNA.^4^ There are three patterns of attachments of small molecules to DNA double helical structures: (i) electrostatic binding results from the interaction of small molecules' positively charged ends with the negatively charged DNA phosphate backbone; (ii) intercalative binding occurs when tiny molecules intercalate within stacked base pairs, changing the DNA shape,^5^ (iii) groove binding: happens in the deep major groove or the shallow minor groove as a result of van der Waals interaction or hydrogen bonding with nucleic acid bases and small molecules. The DNA backbone is either not distorted at all or only slightly by groove binders.^6^ Nonetheless, DNA can directly interact with a wide variety of tiny molecules, and the processes influencing these interactions are quite intricate. Usually, no one approach provides a thorough comprehension of drug–DNA interactions. Consequently, it is crucial to create quick, high-throughput, economical, and ongoing methods to assess these interactions for different medications. These developments can greatly facilitate the processes of drug approval and discovery. According to this viewpoint, DNA is acknowledged as a vital biomacromolecule.

The effectiveness of many drugs is closely linked to their binding mode and affinity to DNA. As a result, studying the intermolecular interactions between small-molecule drugs and DNA has turn out to be a significant focus in the different fields of life sciences, chemistry, and medicine. By reviewing the literature varied drugs were investigated for their DNA binding utilizing fluorescence quenching and molecular docking techniques.^7–11^ Although TAF is primarily recognized for its high-affinity binding to transthyretin protein, its interactions with other essential biomacromolecules, such as DNA, remain largely unexplored. Given that drug–DNA interactions can influence gene expression, mutagenicity, or potential off-target effects, it is valuable to assess whether TAF can interact with DNA and to characterize the nature of such binding. Studying its DNA-binding behavior can reveal off-target effects, provide insight into its safety profile, and contribute to understanding its broader pharmacological interactions. Therefore, the aim of this study is to systematically investigate the binding interaction between TAF and calf thymus DNA, for the first time, using a multimodal approach, including UV-visible and fluorescence spectroscopy, viscosity measurements, ionic strength effects, thermodynamic analysis, and computational modeling. The results of this study may enhance the understanding of TAF's interaction landscape and contribute to future drug safety and mechanism-of-action studies.

## 2. Experimental

### 2.1. Materials, reagents and methods

Analytical grade chemicals were purchased and utilized directly without extra purification. Calf thymus DNA (ct-DNA) was bought from Sigma Aldrich (St. Louis, MO, USA). A 0.05 M Tris–HCl buffer solution was made by dissolving 0.78 g in 100.0 mL of distilled water, with 1 M HCl employed to adjust the pH to 7.4. A stock solution of ct-DNA was prepared in Tris–HCl buffer by dissolving 0.01 g of ct-DNA and diluting to a final volume of 50.0 mL with the buffer. The solution was subsequently stored at 4 °C in dark for duration of up to 7 days. The ct-DNA solution was sonicated prior to the tests to guarantee solution homogeneity. The purity of DNA was assessed by measuring the UV absorbance ratio of *A*_260_/*A*_280_, which exceeded 1.9, indicating that the DNA was suitable for experimental purposes.^12,13^ Distilled water was used whenever needed in the study.

Tafamidis was purchased from Pfizer, New York, NY 10017, USA. Ethanol was used to prepare the stock solution of TAF, which had a final concentration of 1.0 × 10^−3^ M. We purchased ethidium bromide (EB), rhodamine B (RB), Tris–HCl from Sigma Aldrich (St. Louis, MO, USA). The solutions of RB (2.0 × 10^−3^ M) and EB (1.2 × 10^−3^ M) were prepared by dissolving in ethanol and stored at 4 °C.

### 2.2. Instruments

• T80+ UV/vis PC spectrophotometer (PG Instruments Ltd, Woodway Lane, Wibtoft, England) with a 1.0 cm quartz cell.

• Spectrofluorimetric measurements were conducted using a Cary Eclipse spectrofluorimeter with a xenon flash lamp (Agilent Technologies, Santa Clara, CA, USA).

• An Oswald viscometer, maintained at a controlled temperature of 298 K, was utilized in the viscosity measurements.

### 2.3. Techniques for studying the binding interaction

#### 2.3.1. Spectrophotometric measurements

Standard procedures for drug–DNA interaction studies *via* UV-vis spectroscopy were followed as described in earlier literature.^14,15^ Using T80+ UV/vis PC spectrophotometer; the UV spectra of 3.0 × 10^−5^ M ct-DNA solutions were recorded within the 200–400 nm range while gradually increasing TAF concentration (from 0 to 1.5 × 10^−5^ M). Baseline correction was performed using appropriate blanks (buffer or solvent only), and all measurements were conducted at least in triplicate. Measurements were conducted accurately at three temperatures (298, 303, and 308 K) to assess binding constants and assess the influence of temperature on the drug–DNA interaction. Furthermore, the absorbance of a solution containing TAF (0.9 × 10^−5^ M) and ct-DNA (3.0 × 10^−5^ M) was analyzed at 298 K under varying NaCl concentrations (0 to 0.07 M) to assess the influence of strength of ions on interaction. NaCl solutions of various concentrations were freshly prepared and added to the DNA–TAF complex to assess ionic strength effects. Absorbance changes were monitored after equilibration at 298 K.^16^ All absorbance values were measured at 260 nm.

#### 2.3.2. Viscosity measurements

An Oswald viscometer (a capillary inner diameter of 0.57 mm), was used for viscosity measurements, thermostated at 298 K by means of a fixed-temperature bath. The ct-DNA concentration was maintained constant at 30.0 μM in a Tris–HCl buffer solution (pH 7.4), with varying TAF concentrations (0–15.0 μM). A digital stopwatch was employed to accurately compute the flow time of the solutions. Each viscosity measurement was repeated three times to ensure reproducibility, and the average flow time was recorded. The relative specific viscosity was calculated using established methods.^17,18^ Hence, the relative specific viscosity (*μ*/*μ*_0_)^1/3^ was calculated based on the average values obtained from the three independent replicate measurements. Here, *μ*_0_ represents the specific viscosity of the ct-DNA solution, while *μ* denotes the specific viscosity of the ct-DNA–TAF complex. The viscosity measurements were executed to assess the structural changes in DNA on interaction with TAF, providing insights into the binding mode. The findings were analyzed by plotting (*η*/*η*_0_)^1/3^ as a function of the binding ratio *r* (*r* = [TAF]/[ct-DNA]), allowing for a quantitative evaluation of the impact of TAF on the DNA's hydrodynamic properties.

#### 2.3.3. Spectrofluorimetric measurements

Standard procedures for drug–DNA interaction studies *via* fluorescence spectroscopy were followed as described in earlier literature.^14,15^ Mixtures containing ct-DNA (30.0 μM) were measured in the existence of either of the fluorescent probes. The fluorescence measurements were conducted to examine the binding interactions between ct-DNA and the probes, offering insights into structural and environmental changes upon interaction. These probes are RB (2.0 × 10^−3^ M, *λ*_ex_/*λ*_em_ = 465/577 nm) and EB (1.2 × 10^−3^ M, *λ*_ex_/*λ*_em_ = 525/602 nm), which correspond to different groove and intercalation binding probes, respectively, in the presence and absence of varying TAF concentrations (0–15.0 μM). Three measurements of each emission spectrum were made, and the average reading was calculated.

#### 2.3.4. Molecular docking

Molecular docking studies with TAF and two calf thymus B-DNA sequences were executed by using AutoDock 4.2 software. The 3D coordinates of the calf thymus B-DNA sequences (PDB ID: 1D29 and 3EY0) were acquired from the Protein Data Bank and TAF structure from the PubChem database. The B-DNA was prepared for docking by adding the Kollman charges and saving in .pdbqt format. The ligand is energy reduced utilizing MMFF94 force-field in Chem3D ultra 16.0 software.^19,20^ The autogrid function was set up with the grid box dimensions at a spacing point of 0.375 as mentioned in Table 1. The Lamarckian genetic algorithm was investigated for binding energy calculation with a population size of 100 and iteration of 2.5 million times. The visualization of 3D and 2D interacting diagram for the presentation for TAF–calf thymus DNA complexes were obtained by Chimera 1.14 version and Biovia Discovery Studio software, respectively.^21,22^

**Table 1:** PDB entries and grid point data for the two DNA sequences

DNA (PDB ID)	Sequence	Grid points (coordinates) *x*, *y*, *z*	Grid points (sizes) *x*, *y*, *z*	Population size	Iteration (million)
3EY0	5′-(ATA TAT ATAT)-3′	16.418, 10.417, 90.217	100, 62, 94	150	2.5
1D29	5′-(CGT GAA TTC ACG)-3′	14.92, 20.905, 8.82	72, 72, 126	150	2.5

#### 2.3.5. Molecular dynamic simulation

Molecular dynamics (MD) simulation was performed using GROMACS 2022.2. The simulations were executed in a three-stage process.

##### 2.3.5.1. Enzyme preparation

The three-dimensional structures (3D) of the ligand–DNA complexes were saved in .pdb format by PyMOL software for further analysis. Molecular dynamics simulations (MD) were performed utilizing the GROMACS software package (version 2022.2) to analyze the dynamic behavior of the complexes. The simulations afforded atomic-level insights into the binding mechanism and structural stability of the TAF–DNA complex.^23–25^ The DNA topology was created with the AMBER99SB force field,^26^ while the ligand topology was prepared utilizing the SwissParam server.^27^

##### 2.3.5.2. Developing a system for simulation

Following the application of the force field, the DNA–drug complexes were incorporated into the system and solvated using the TIP3P water model within a cubic box, maintaining a minimum distance of 1 nm between the DNA and the box edges.^28^ Periodic boundary conditions were imposed, beside neutralization of the system with Na^+^ ions. Energy minimization was performed for 50 000 steps using the steepest descent algorithm, subsequently a 100 ps *NVT* simulation at 300 K and a 100 ps *NPT* simulation for system equilibration. The leapfrog algorithm was applied within the constant-temperature and pressure system (*NPT*) ensemble to independently couple the DNA, ligand, water molecules, and ions.^29^ To ensure a stable environment at 300 K and 1 bar pressure, the Berendsen temperature and pressure coupling constants were set to 0.1 and 2, respectively.^30^ Subsequently, a 100 ns molecular dynamics (MD) simulation was investigated under isothermal–isobaric conditions at 300 K. A pressure coupling time constant of 1 ps was applied to keep a stable pressure of 1 bar, while the LINCS algorithm was employed to constrain bond lengths.^31^ van der Waals and Coulomb interactions were shortened at 1.2 nm, and the Particle Mesh Ewald (PME) method^32^ implemented in GROMACS was utilized to reduce errors associated with interaction truncations.^33–35^

##### 2.3.5.3. Analysis and visualization of simulation

The trajectory files were examined using VMD (Visual Molecular Dynamics) 1.9.2 (ref. 36) and analyzed with the custom-developed tool HeroMDAnalysis^37,38^ and Xmgrace 5.1.25.^39^

## 3. Results and discussion

Understanding the binding interactions among drug molecules and DNA has provided insights into the drug's binding mode. Consequently, DNA–ligand binding studies play a vital role in the improvement of molecular probes and therapeutic agents, guiding the design and synthesis of new drugs. Owing to the importance of TAF in treatment of different diseases, this work investigates the interaction between TAF and ct-DNA using multi-spectroscopic techniques. By analyzing the binding mode, affinity, and interaction forces, the findings offer insights into TAF's molecular behavior, contributing to its understanding in drug development, industry and therapeutic applications.

### 3.1. UV-vis absorption spectra and binding constant determination

**Fig. 2:**
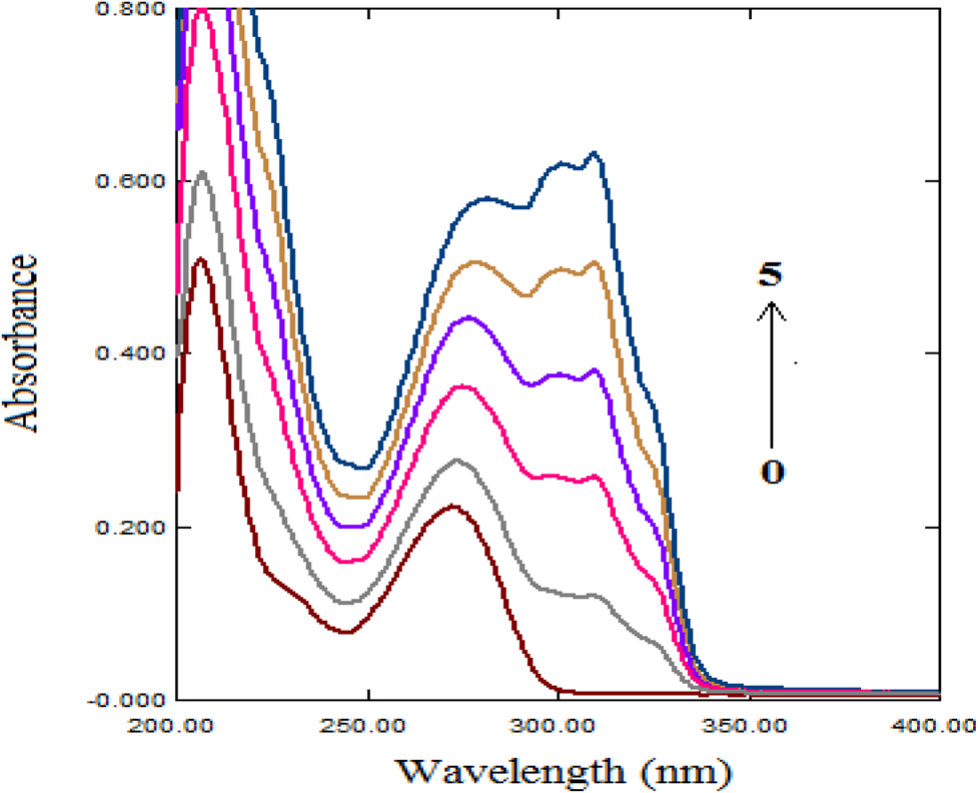
UV absorption spectra of ct-DNA (3.0 × 10^−5^ M) with different concentrations of TAF. The concentrations of TAF from 0 to 5 were 0, 0.3 × 10^−5^, 0.6 × 10^−5^, 0.9 × 10^−5^, 1.2 × 10^−5^, and 1.5 × 10^−5^ M, respectively.

The interaction between TAF and ct-DNA was initially assessed using UV-vis absorption spectroscopy by monitoring spectral changes of DNA at 260 nm upon incremental addition of TAF. As shown in Fig. 2, a distinct hyperchromic effect was observed with no significant shift in the maximum absorbance wavelength. This observation suggests that TAF binds to DNA through non-intercalative mechanisms, as intercalative binding is usually associated with bathochromic or hypsochromic shifts due to π–π stacking interactions that alter the electronic environment of the DNA base pairs. The increase in absorbance (hyperchromicity) indicates partial unwinding or loosening of the double helix, which supports a groove-binding mode, particularly within the minor groove. Groove-binding agents typically cause slight spectral changes due to local conformational alterations without major disruption of base stacking, in contrast to classical intercalators such as ethidium bromide.^14,15,17^

The efficacy of a drug is closely linked to its binding affinity, making the study of its interaction with biomacromolecules essential. Binding affinity can be assessed using either the binding constant (*K*_b_) or the dissociation constant (*K*_d_). To quantitatively evaluate the binding strength between TAF and ct-DNA, the binding constant (*K*_b_) was calculated using the Benesi–Hildebrand eqn (1), which is commonly applied to 1 : 1 ligand–DNA interactions:^15,17^1
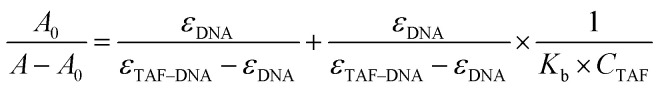
As: *A* and *A*_0_ denote the absorbances of ct-DNA with and without the drug, respectively. *ε*_DNA_ and *ε*_TAF–DNA_ represent the molar extinction coefficients of ct-DNA and the drug–ct-DNA complex, respectively. *C*_TAF_ corresponds to the concentration of TAF.

**Fig. 3:**
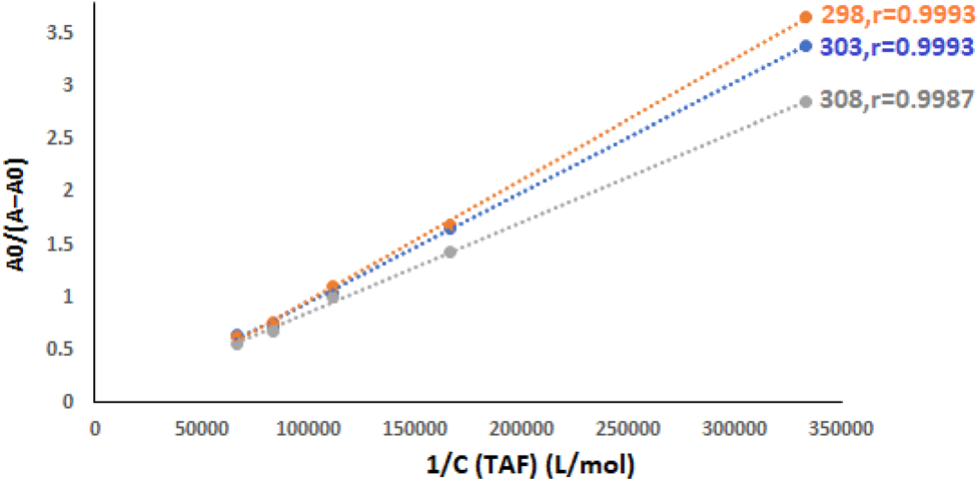
Plot of *A*_0_/(*A* − *A*_0_) against 1/*C*_TAF_ at different temperature settings (*C*_DNA_ = 3.0 × 10^−5^ M), while *r* is the correlation coefficient.

The absorbance values were fitted to the Benesi–Hildebrand equation, and plots of *A*_0_/(*A* − *A*_0_) *versus* 1/[TAF] yielded linear relationships at different temperatures (298, 303, and 308 K) as presented in Fig. 3. These linear plots confirm a 1 : 1 binding stoichiometry between TAF and ct-DNA. The calculated binding constants (*K*_b_) were on the order of 10^5^ M^−1^ (Table 2), which indicates a moderate-to-strong binding affinity, comparable to other known minor groove binders.^7^ These values are lower than those typically reported for intercalators such as ethidium bromide (*K*_b_ ≈ 10^6^ M^−1^),^40,41^ further supporting the conclusion that TAF interacts with ct-DNA *via* minor groove binding.

**Table 2:** Estimation of the binding constants (*K*_b_) at three varied temperatures and thermodynamic parameters of ct-DNA–TAF complex

*T* (K)	*K*_b_ (M^−1^)	Δ*H*° (kJ mol^−1^)	Δ*S*° (J mol^−1^ K^−1^)	Δ*G*°^a^ (kJ mol^−1^)	Δ*G*°^b^ (kJ mol^−1^)
298	2.2 × 10^5^	6.23	123.48	−30.57	−30.47
303	2.4 × 10^5^			−31.17	−31.18
308	2.5 × 10^5^			−31.80	−31.88

a Δ*G*° = *RT* ln *K*_b_.

b Δ*G*° = Δ*H*° − *T*Δ*S*°.

These spectroscopic observations, along with the calculated binding constants and absence of major spectral shifts, suggest that the interaction between TAF and ct-DNA is characterized by a specific and stable minor groove binding mode, rather than intercalation or electrostatic attraction. This conclusion is consistent with the findings from viscosity studies, fluorescence displacement assays, and molecular docking simulations discussed in the following sections.

### 3.2. Viscosity measurements

While UV-vis spectroscopy provides initial insight into the mode of interaction, it alone cannot definitively distinguish between groove binding and intercalation. Therefore, to further validate the proposed non-intercalative (groove) binding of TAF to ct-DNA, its effect on the viscosity of DNA solutions was investigated.^18^ Viscosity measurements are a classical method for probing DNA structural changes: intercalative binding increases viscosity by unwinding the helix to accommodate small molecules, while non-classical intercalation reduces viscosity by twisting and shortening the helix, whereas groove binding or electrostatic interactions typically result in negligible changes.^15,17,18,42^ Hence, viscosity studies serve as a complementary tool to clarify the binding mode suggested by spectroscopic analysis.

**Fig. 4:**
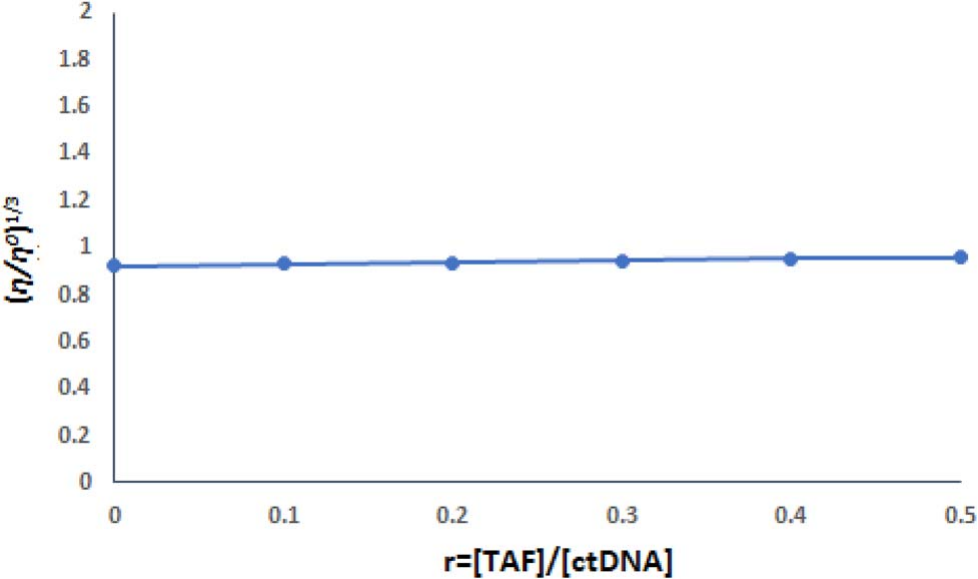
Influence of variable concentrations of TAF (0–15.0 μM) on the viscosity of ct-DNA (30.0 μM) in Tris–HCl buffer.

The findings indicated that the relative specific viscosity (*η*/*η*_0_)^1/3^ values of ct-DNA largely remained stable with rising TAF concentrations (Fig. 4), implying that the interaction between TAF and ct-DNA occurs through a groove binding mechanism. This aligns with the results of spectrophotometric analyses.

### 3.3. Competitive spectrofluorimetric study for determination of binding sites

This method utilizes well-characterized DNA-binding probes, such as rhodamine B (RB) or ethidium bromide (EB), which exhibit a notable fluorescence increase upon interaction with DNA.^43,44^ Studies have shown that EB binds by intercalating between DNA base pairs, whereas RB primarily associates with the minor groove of DNA, favoring AT-rich sequences.^16,45^

This study examined how TAF interacts with DNA when two different fluorescent probes (EB and RB) are already bound to the DNA. When TAF was added to the DNA–EB complex, there was little change in the fluorescence of the probe, indicating that the drug and EB do not compete for binding sites on the DNA. However, when TAF was added to the DNA–RB complex, the fluorescence of RB gradually decreased. This suggests that the studied drug competes with RB for binding to the smaller groove of the DNA molecule.

**Fig. 5:**
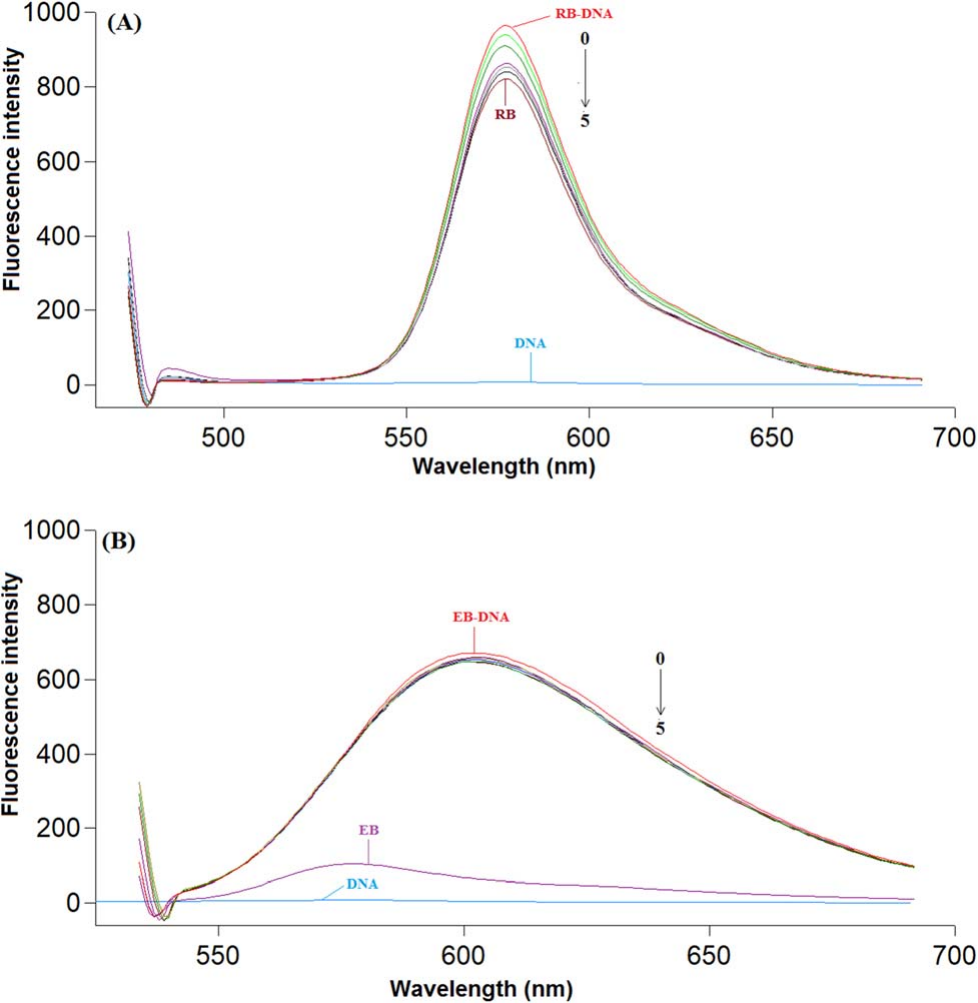
(A) Fluorescence emission spectra of the RB–ct-DNA complex in presence and absence of TAF at 298 K. *C*(ct-DNA): 30.0 μM; *C*(RB): 2.0 × 10^−3^ M; *C*(TAF): (0 → 5): 0, 3.0, 6.0, 9.0, 12.0, 15.0 μM (*λ*_ex_/*λ*_em_ = 465/577 nm). (B) Fluorescence emission spectra of the EB–ct-DNA complex in presence and absence of TAF at 298 K. *C*(ct-DNA): 30.0 μM; *C*(EB): 1.2 × 10^−3^ M; *C*(TAF) (0 → 5): 0, 3.0, 6.0, 9.0, 12.0, 15.0 μM (*λ*_ex_/*λ*_em_ = 525/602 nm).

These findings support the idea that TAF binds to DNA by fitting into the smaller groove, rather than inserting itself between the DNA base pairs. This assumption is consistent too with the findings obtained from the spectrophotometric and viscosity studies (Fig. 5).

These results not only reinforce the hypothesis of TAF binding in the minor groove but also indicate a preferential interaction with AT-rich sequences, consistent with the known binding preferences of groove-targeting ligands.

### 3.4. Influence of ionic strength

**Fig. 6:**
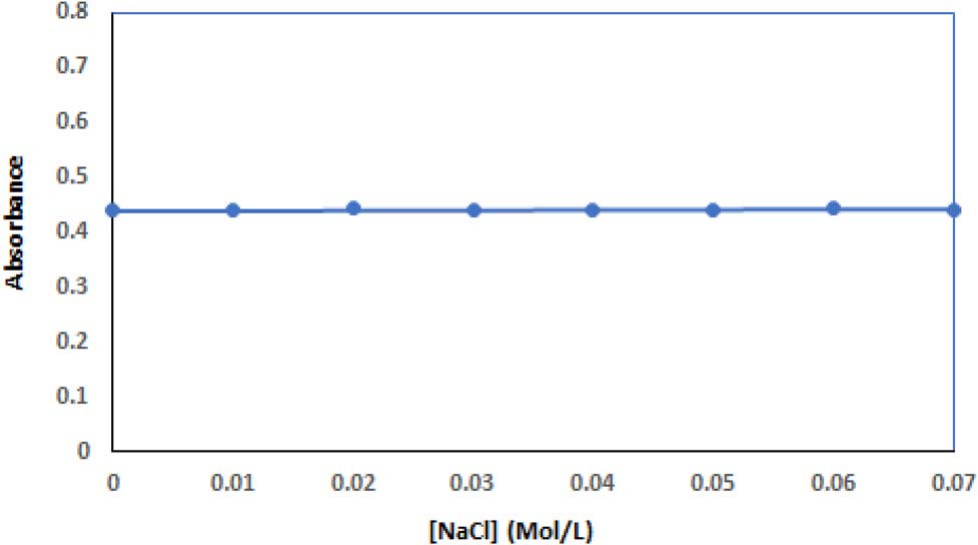
Effect of NaCl ionic strength on the absorbance of ct-DNA–TAF complex. Concentrations of TAF and ct-DNA were 0.9 × 10^−5^ M and 3.0 × 10^−5^ M, respectively. NaCl concentration was raising from 0 to 0.07 M.

The strength of electrical forces between molecules is significantly weakened in environments with high salt concentrations, like those found in living organisms. To determine if these forces played a part in the interaction between a ligand (TAF) and DNA (ct-DNA), NaCl concentration was increased from 0 to 0.07 M, as illustrated in Fig. 6. It was found that the ct-DNA–TAF complex's absorbance value was somewhat steady signifying that electrical forces are not the primary factor driving this interaction. Instead, the data indicates that TAF is likely binding to a specific groove within the DNA structure.^46^

### 3.5. Evaluation of thermodynamic parameters and major interaction forces

Hydrogen bonding, electrostatic interactions, hydrophobic forces, and van der Waals forces are the four primary types of non-covalent interactions known to mediate binding between biomacromolecules and small molecules. Hence, these forces play a critical role in deciding the specificity, stability, and strength of molecular interactions.^47^ Additionally, one can deduce the type of binding forces by examining both the sign and value of entropy (Δ*S*°) and enthalpy (Δ*H*°) values. It is commonly considered that van der Waals force and/or hydrogen bonding are the fundamental contact forces when both Δ*S*° and Δ*H*° are negative. The main attraction is hydrophobic when both Δ*S*° and Δ*H*° are positive, or electrostatic when Δ*S*° is positive and Δ*H*° is close to zero.^48,49^ The thermodynamic parameters, such as Gibbs free energy change (Δ*G*°), Δ*S*°, and Δ*H*° in the interaction of TAF with ct-DNA were computed using Van't Hoff eqn (2) and (3):^49^2
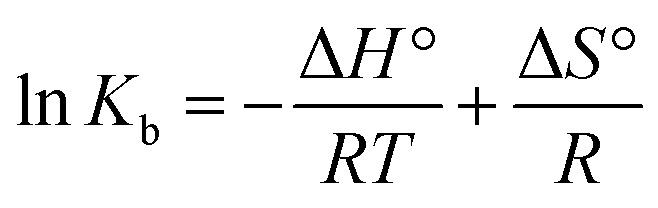

3 Δ*G*° = Δ*H*° − *T*Δ*S*°

where, *R* represents a gas constant.

**Fig. 7:**
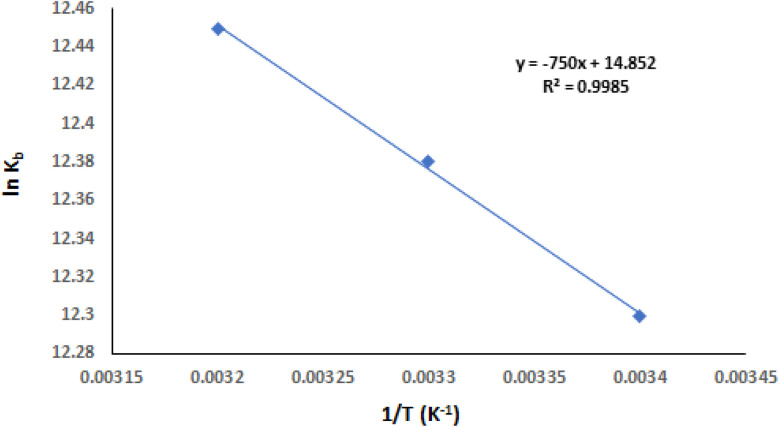
Van't Hoff plot for the ct-DNA–TAF complex.

Using the slope and intercept of the Van't Hoff plot of ln *K*_b_*vs.* 1/*T* (Fig. 7), the values of Δ*H*° and Δ*S*° were computed and are shown in Table 2. It is found that there is a spontaneous binding interaction between ct-DNA and TAF since Δ*G*° is less than zero. From the obtained results, the positive Δ*H*° and Δ*S*° values suggest that hydrophobic interactions dominate the binding process. Such interactions are typical of minor groove binders, which often rely on shape complementarity and van der Waals forces, rather than classical ionic or stacking interactions. This thermodynamic profile supports the fluorescence and docking results and contributes to the overall understanding of TAF's biophysical behavior.

While the spectroscopic and thermodynamic data collectively suggest that TAF interacts with ct-DNA primarily through a minor groove binding mode driven by hydrophobic forces, these techniques cannot directly reveal the precise atomic-level interactions or spatial conformation of the ligand within the DNA structure. Therefore, to gain deeper molecular insight into the binding mechanism, computational approaches were employed, including molecular docking and molecular dynamics (MD) simulations. These theoretical tools complement the experimental findings by visualizing the TAF–DNA complex, identifying specific interaction sites, and assessing the dynamic behavior and stability of the complex over time.

### 3.6. Molecular modeling

**Fig. 8:**
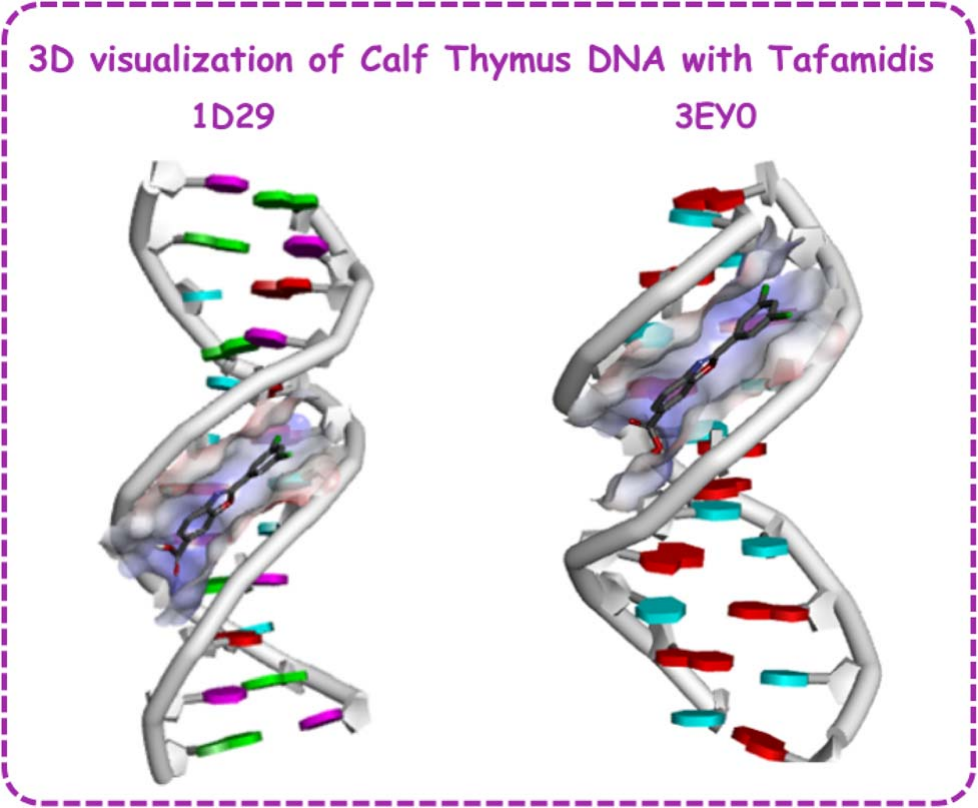
3D visualization of tafamidis–calf thymus DNA complex.

**Fig. 9:**
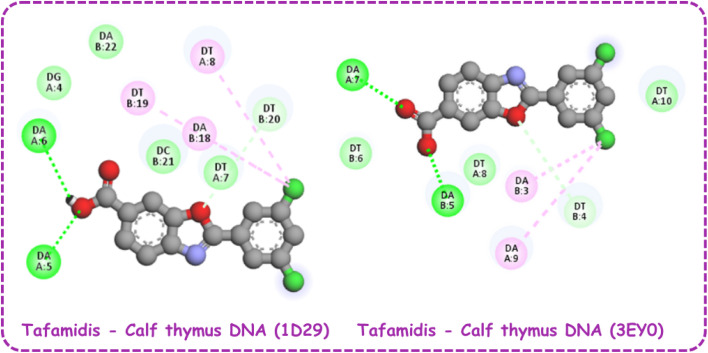
2D visualization of tafamidis–calf thymus DNA complex.

Docking studies of TAF with two calf thymus B-DNA sequences, identified by PDB IDs 3EY0 and 1D29, provide key insights into its binding behavior and interaction^7,9,10^ (Table 3). The 3D and 2D visualization of the TAF–calf thymus DNA complex is provided in Fig. 8 and 9. The binding affinities were determined to be −7.75 kcal mol^−1^ for 1D29 and −7.61 kcal mol^−1^ for 3EY0, indicating a slightly stronger interaction with the B-DNA represented by 1D29. Tafamidis demonstrates conformational flexibility due to three active torsional bonds namely C9–C11, C19–C20, and C20–O21. These torsional movements enable the molecule to adapt its conformation to fit the curvature of the DNA minor groove, ensuring stable and specific binding. This alignment is essential for establishing effective hydrogen bonds and hydrophobic interactions with DNA bases, contributing to the stability of the TAF–DNA complexes. For 1D29, hydrogen bonds are formed with adenine residues DA6 and DA5, while hydrophobic interactions include pi–alkyl bonds with DT19 and DT8 and a C–H bond with DT20. Similarly, in 3EY0, hydrogen bonds are observed with DA5 and DA7, while hydrophobic interactions include a C–H bond with DT4 and pi–alkyl bonds with DA3 and DA9.

**Table 3:** Binding affinity and interaction of TAF with calf thymus DNA sequence

PDB ID	Binding affinity (kcal mol^−1^)	Interaction sites	
		Hydrogen bonds	Hydrophobic bonds
1D29	−7.75	DA6, DA5	DT19, DT8 (pi–alkyl), DT20 (C–H bond)
3EY0	−7.61	DA5, DA7	DT4 (C–H bond), DA3, DA9 (pi–alkyl)

The estimated binding free energies (Δ*G*) are consistent with the docking results. The intermolecular energy (*E*_1_), representing contributions from van der Waals forces, hydrogen bonds, desolvation, and electrostatic interactions, is −8.64 kcal mol^−1^ for 1D29 and −8.50 kcal mol^−1^ for 3EY0, indicating favorable interactions. Internal strain energy (*E*_2_) values are minimal (−0.28 kcal mol^−1^ for 1D29 and −0.29 kcal mol^−1^ for 3EY0), suggesting minor conformational changes upon binding. The torsional free energy (*E*_3_) remains constant at 0.89 kcal mol^−1^, while the unbound system energy (*E*_4_) values are identical for both complexes (−0.28 kcal mol^−1^ and −0.29 kcal mol^−1^). The binding free energies exhibited by the TAF–DNA complex are provided in Table 4. These findings highlight the ligand's ability to establish stable and energetically favorable interactions with both B-DNA sequences.

**Table 4:** Theoretical binding energies of the TAF with calf thymus DNA sequence complex involved in docking^a^

PDB ID	Δ*G* (kcal mol^−1^)	*E*_1_ (kcal mol^−1^)	*E*_2_ (kcal mol^−1^)	*E*_3_ (kcal mol^−1^)	*E*_4_ (kcal mol^−1^)
1D29	−7.75	−8.64	−0.28	0.89	−0.28
3EY0	−7.61	−8.50	−0.29	0.89	−0.29

a Δ*G* – total binding free energy estimated (*E*_1_ + *E*_2_ + *E*_3_ − *E*_4_); *E*_1_ – total intermolecular energy (sum of van der Waals, hydrogen bond, desolvation, electrostatic energies); *E*_2_ – total internal energy; *E*_3_ – torsional free energy; *E*_4_ – unbound system energy.

Although this study did not directly assess the biological consequences of TAF binding to ct-DNA, the observed minor groove binding to AT-rich regions may have implications for gene regulation and DNA-associated processes. Minor groove binders are known to interfere with transcription factor binding, RNA polymerase progression, or replication machinery, depending on their binding location and affinity. Given TAF's preferential interaction with DNA without major structural distortion, it is plausible that subtle regulatory interference could occur at specific genomic loci. Further studies using cell-based models, gene expression profiling, or DNA polymerase assays would be necessary to determine whether TAF impacts gene transcription or replication under physiological conditions.^50^

### 3.7. Molecular dynamic simulations

To evaluate the binding interactions of TAF with B-DNA (PDB ID: 1D29), molecular dynamics (MD) simulations were established for 100 nanoseconds.^8,9^ The simulation data was analyzed utilizing different statistical values, including Root-Mean-Square Deviation (RMSD), Root-Mean-Square Fluctuation (RMSF), hydrogen bond interactions, the percentage occupancies of these interactions, radius of gyration (*R*_g_), and Solvent Accessible Surface Area (SASA) over the simulation period.

#### 3.7.1. RMSD analysis

**Fig. 10:**
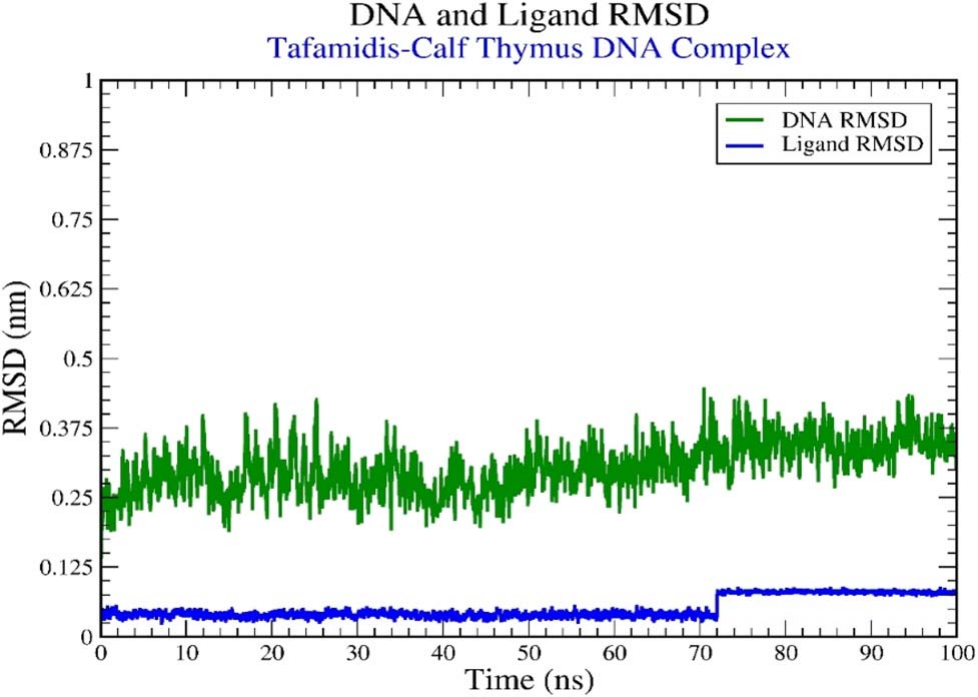
Graphical representation of the plots displaying DNA RMSD (nm) against time (100 ns) for tafamidis–calf thymus DNA (1D29) complex.

The RMSD analysis offered crucial insights into the structural stability of the DNA–ligand complex. As illustrated in Fig. 10, the RMSD values of the DNA remained consistent throughout the simulation, ranging between 0.2 and 0.4 nm, suggesting minimal structural deviations and a stable configuration. The ligand's RMSD was also analyzed to determine its binding stability to the DNA. The ligand exhibited stable RMSD values between 0.025 and 0.1 nm, reflecting a strong and reliable interaction with the B-DNA of calf thymus. A minor fluctuation was observed at 72 ns, where the RMSD increased to 0.75 nm, indicating slight conformational adjustments to accommodate the minor groove of the DNA.

#### 3.7.2. RMSF analysis

**Fig. 11:**
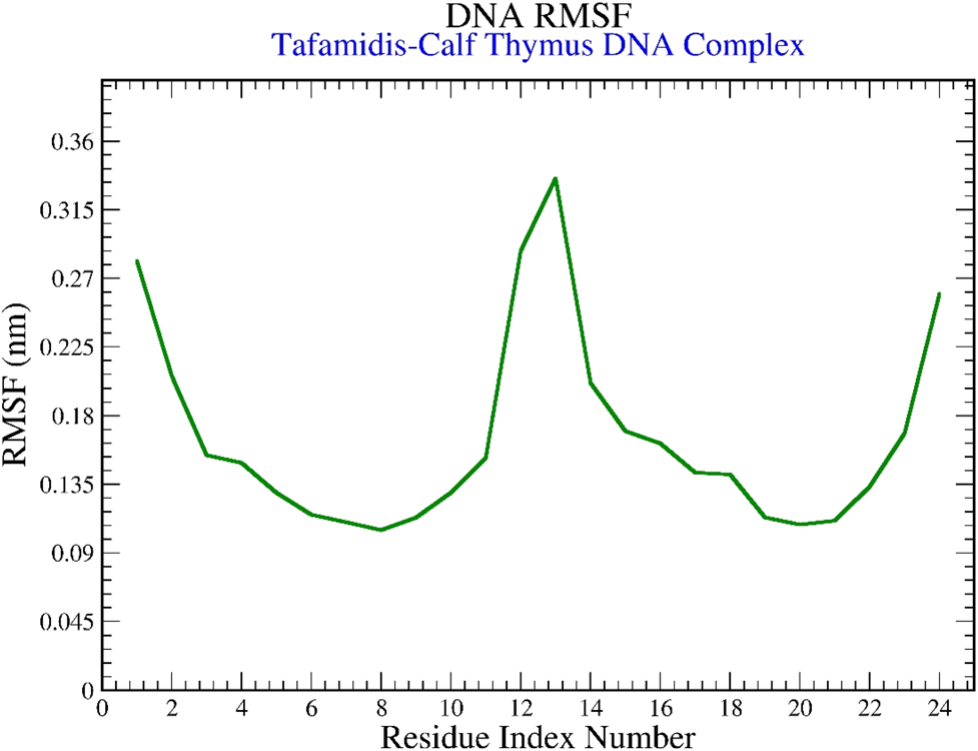
Graphical representation of the plots displaying the DNA RMSF (nm) *versus* residue index number of tafamidis–calf thymus DNA (3EY0) complex.

The RMSF analysis, presented in Fig. 11, provided details on localized fluctuations within the DNA structure. The plot showed that most residues fluctuated within the range of 0.1 to 0.3 nm. However, residues around positions 11 to 14 displayed slightly higher fluctuations, peaking at 0.35 nm. These findings highlight the overall structural stability of the B-DNA during the simulation, with localized flexibility in specific regions.

#### 3.7.3. H-bond interaction

**Fig. 12:**
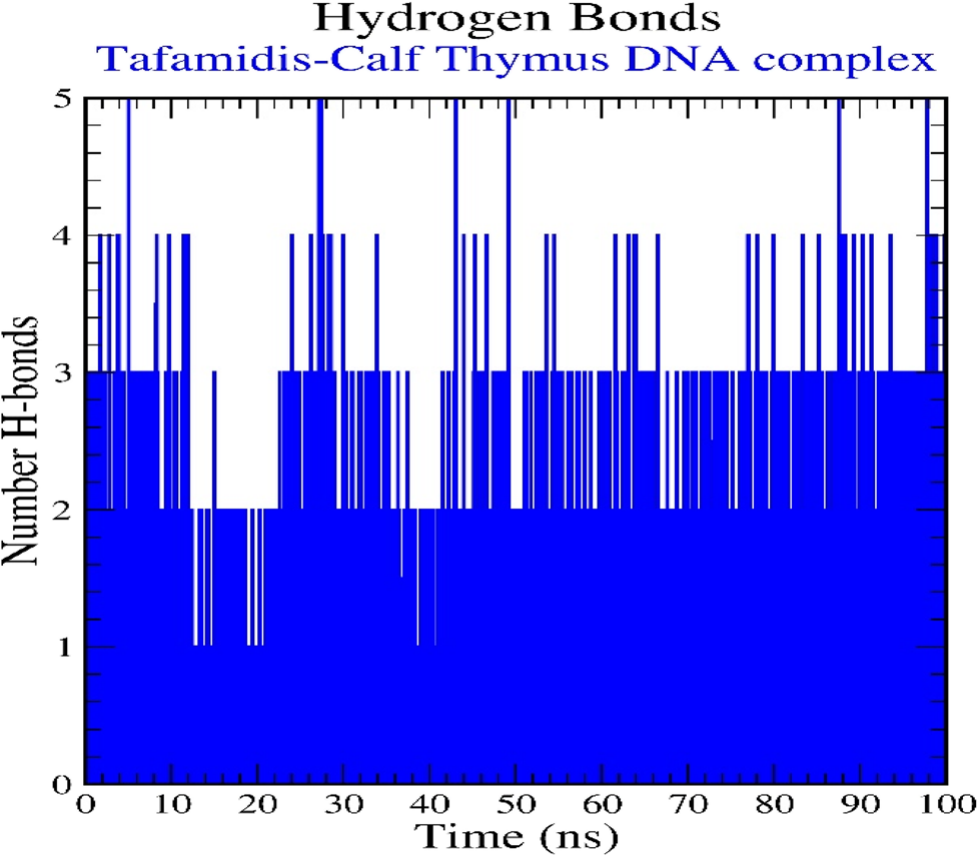
Pictorial representation of the number of H-bond contacts formed by tafamidis–calf thymus DNA (1D29) complex.

**Fig. 13:**
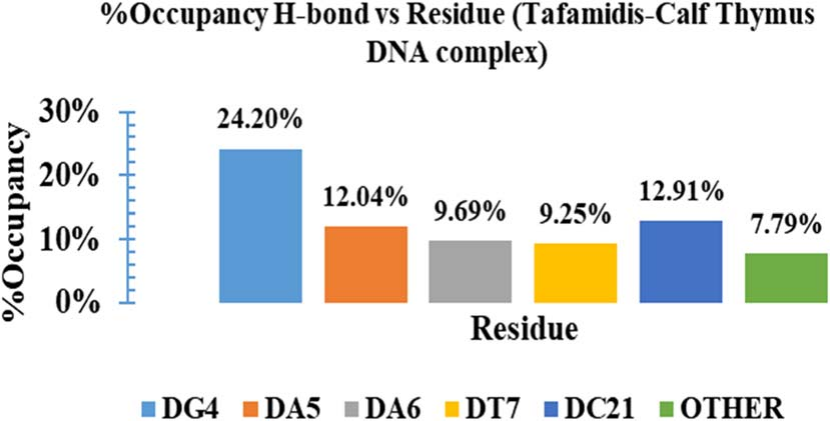
Histogram representation of % occupancies of the H-bond DNA–ligand contacts of tafamidis–calf thymus DNA (1D29) complex.

Hydrogen bond interactions, a vital factor in the stability of the DNA–ligand complex, were also evaluated. As shown in Fig. 12, TAF consistently formed an average of two hydrogen bonds with the DNA throughout the simulation. The percentage occupancies of these hydrogen bonds, detailed in Fig. 13, revealed that TAF exhibited the strongest interaction with residue DG4, with an occupancy of 24.20%. The significant interactions were also observed with adenine residues DA5 and DA6, showing occupancies of 12.04% and 9.69%, respectively, and thymine residue DT7, with an occupancy of 9.25%. These findings confirm the formation of stable hydrogen bonds these residues during the simulation, emphasizing TAF's strong and stable binding to the AT-region of DNA.

#### 3.7.4. MMGBSA analysis

**Fig. 14:**
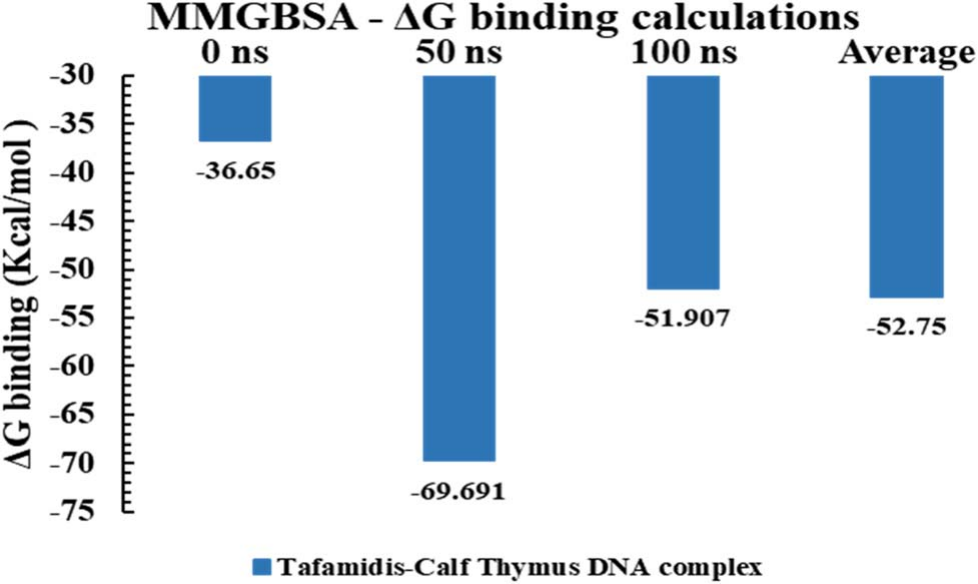
MMGBSA Δ*G* binding energy calculations for tafamidis–calf thymus DNA (3EY0) complex.

The binding free energy Δ*G* values for the TAF–calf thymus DNA complex (PDB ID: 1D29) were analyzed at different time points during the molecular dynamics simulation (0 ns, 50 ns, and 100 ns) to evaluate the stability and strength of the interaction (Table 5 and Fig. 14). At the beginning of the simulation (0 ns), the complex exhibited a binding free energy of −36.65 kcal mol^−1^, signifying a stable interaction. By 50 ns, the binding free energy significantly decreased to −69.691 kcal mol^−1^, reflecting a stronger and more favorable interaction, likely due to conformational adjustments optimizing the ligand's fit within the DNA's minor groove. At 100 ns, the binding free energy increased slightly to −51.907 kcal mol^−1^, suggesting some conformational relaxation while maintaining a strong binding interaction. The average binding free energy over the simulation period was −52.75 kcal mol^−1^, confirming a consistently favourable and stable interaction between TAF and the DNA. These results demonstrate the ligand's ability to adapt its conformation over time to maintain robust binding with the DNA target.

**Table 5:** MMGBSA Δ*G* binding energy calculations for TAF–calf thymus DNA (3EY0) complex

Ligand–DNA complex	0 ns	50 ns	100 ns	Average
TAF–calf thymus DNA complex (1D29)	−36.65	−69.691	−51.907	−52.75

#### 3.7.5. Radius of gyration (*R*_g_)

**Fig. 15:**
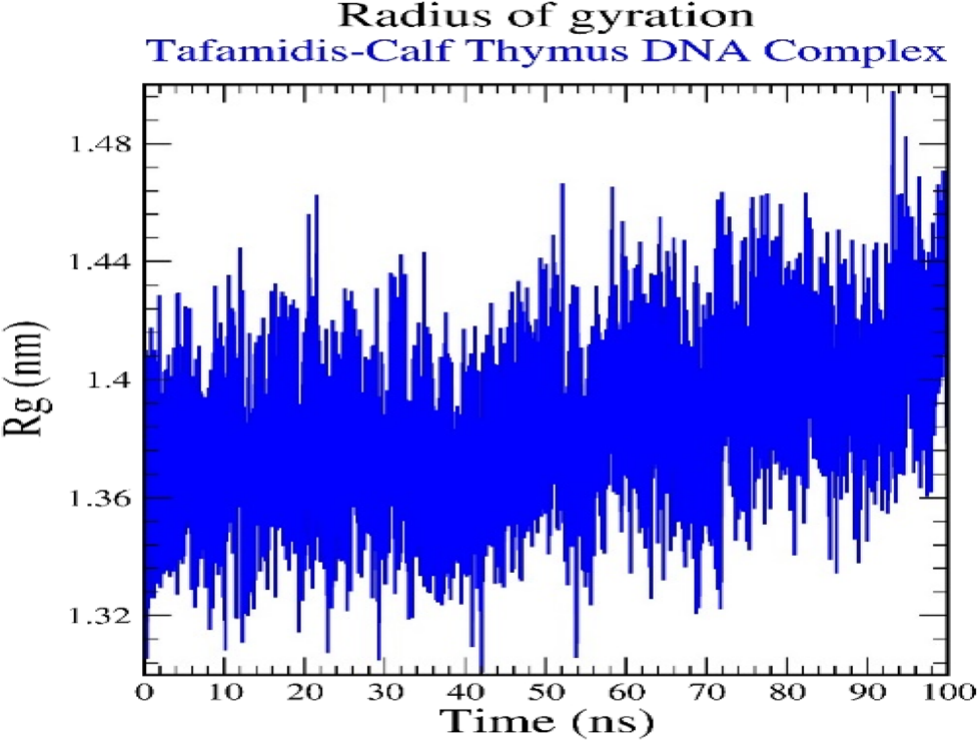
Radius of gyration for tafamidis–calf thymus DNA (1D29) complex.

The plot of the radius of gyration (*R*_g_) *versus* time provides insights into the structural compactness and stability of the TAF–calf thymus DNA complex during the 100 ns molecular dynamics simulation (Fig. 15). The *R*_g_ values range between approximately 1.32 nm and 1.48 nm, indicating consistent structural integrity. Initially, the *R*_g_ values remain stable with minor fluctuations, suggesting that the complex maintains its compactness. However, towards the latter part of the simulation (after 80 ns), there is a slight increase in *R*_g_, indicating a minor expansion of the complex. This could reflect small conformational changes within the DNA–ligand complex as it adapts to stabilize the interaction. Overall, the relatively stable *R*_g_ values throughout the simulation highlight the structural stability and compact nature of the complex.

#### 3.7.6. Solvent accessible surface area (SASA)

**Fig. 16:**
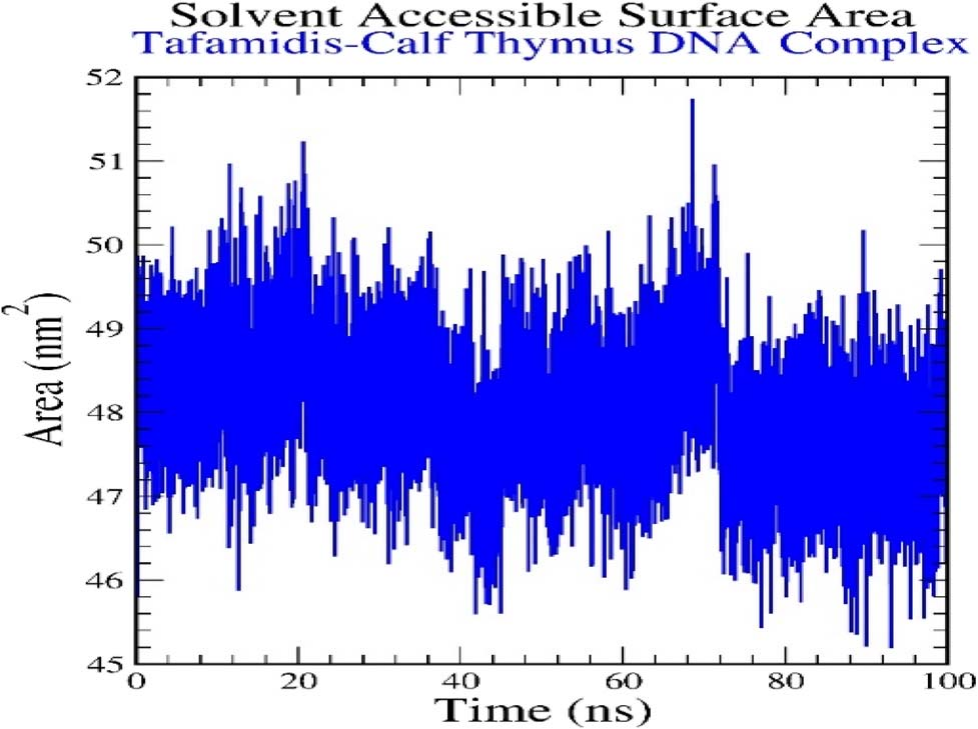
Solvent accessible surface area for tafamidis–calf thymus DNA (1D29) complex.

The plot of Solvent Accessible Surface Area (SASA) *versus* time provides insights into the exposure of the TAF–calf thymus DNA complex to the solvent during the 100 ns molecular dynamics simulation (Fig. 16). The SASA values range between approximately 46 nm^2^ and 51 nm^2^, indicating moderate fluctuations in solvent exposure. Initially, the SASA values fluctuate around 48–50 nm^2^, suggesting that the complex maintains a consistent level of solvent exposure. After 60 ns, there are brief increases and decreases in SASA, reflecting minor conformational adjustments in the complex that temporarily alter the surface area exposed to the solvent. These variations could be attributed to the ligand's movement or slight structural changes in the DNA as it stabilizes the interaction. Overall, the SASA values remain within a narrow range, demonstrating that the TAF–DNA complex maintains its overall structural integrity and interaction stability throughout the simulation, with only minor fluctuations in solvent exposure.

Overall, the MD simulation results confirm the stability and persistence of TAF within the minor groove, highlighting its ability to form stable hydrogen bonds and hydrophobic interactions with AT-rich regions of DNA. This dynamic behavior further validates the experimental conclusions and reveals a consistent binding pattern across methodologies.

Regarding the mechanism of DNA conformational change after TAF binding to ct-DNA, the binding of TAF to ct-DNA does not cause major structural distortion, as evidenced by the viscosity measurements showing negligible changes in the DNA's hydrodynamic length. This supports a minor groove binding mode, which typically induces minimal helical unwinding or extension. Molecular dynamics simulations further clarify the conformational impact: RMSD and RMSF values indicate that the DNA retains overall structural stability with only localized fluctuations, particularly around the binding region (residues 11–14). The radius of gyration (*R*_g_) shows a slight increase toward the end of the simulation, which may reflect minor relaxation or groove widening to accommodate the ligand. Additionally, SASA analysis suggests stable solvent exposure throughout the binding, with no drastic changes in DNA compactness. Together, these findings suggest that TAF binding induces subtle local conformational changes (*e.g.*, minor groove widening or base pair tilting) without disrupting the overall B-form of DNA.

## 4. Study advantages, limitations, and future perspectives

This study provides the first comprehensive investigation of the interaction between TAF and ct-DNA, combining multi-spectroscopic, thermodynamic, and computational modeling techniques. A one major strength of this work is its integrated approach, which enables detailed characterization of the binding mode, interaction forces, and dynamic stability of the TAF–DNA complex. The use of molecular docking and dynamics simulations, supported by experimental data, offers valuable structural insights at the atomic level. From a clinical perspective, our findings may help inform the off-target interaction profile of TAF, particularly in the context of chronic exposure. The observed moderate, minor groove binding to DNA without major structural disruption suggests low genotoxic potential, but also raises important considerations for drug safety and pharmacodynamics. Additionally, understanding such interactions can support regulatory evaluation, especially when considering analogues or modifications of TAF. We believe that this study provides clinically relevant mechanistic insight into TAF behavior at the molecular level, which may be useful in toxicological risk assessment, long-term safety monitoring, and the rational design of next-generation transthyretin stabilizers.

However, the study has some limitations. It should be noted that the present study is limited to equilibrium binding analysis. The kinetics of TAF–DNA interaction (*i.e.*, association and dissociation rate constants) were not assessed. Future investigations using time-resolved spectroscopic or biosensor-based techniques (*e.g.*, surface plasmon resonance) could provide valuable information on the dynamics of this interaction and further elucidate its biological relevance. Moreover, although MD simulations offered detailed insight into the structural dynamics of the TAF–DNA complex, these predictions were not experimentally validated using direct conformational analysis tools such as circular dichroism (CD) spectroscopy or X-ray crystallography. Future work incorporating these techniques could provide experimental confirmation of the localized groove widening and hydrogen-bonding interactions inferred from simulation data, and further clarify the biological significance of TAF–DNA binding. In addition, although TAF is not known to exhibit genotoxic effects clinically, its observed binding to DNA, even through minor groove interaction, suggests that chronic exposure scenarios could merit additional study. Future investigations including genotoxicity assays, DNA repair evaluations, or *in vivo* long-term studies may help clarify whether TAF poses any risk to DNA integrity under repeat dosing conditions.

## 5. Conclusion

The present study offers the first detailed analysis of the interaction between TAF, a clinically relevant drug for transthyretin amyloidosis, and ct-DNA using a comprehensive multimodal approach. UV-vis spectroscopy revealed a hyperchromic effect without a shift in absorption maximum, suggesting a non-intercalative binding mode. This was further supported by viscosity measurements, which showed negligible changes in DNA length, and fluorescence displacement studies that indicated competition with RB but not EB, confirming minor groove binding behavior. The calculated binding constants (∼10^5^ M^−1^) suggest a moderate-to-strong interaction with ct-DNA. Thermodynamic analysis revealed positive Δ*H*° and Δ*S*° values, indicating that hydrophobic interactions are the primary driving force, supported by the lack of salt-dependence in the binding. Molecular docking confirmed TAF's insertion into the DNA minor groove, establishing hydrogen bonds and hydrophobic contacts with AT-rich regions. Molecular dynamics simulations demonstrated the structural stability of the TAF–DNA complex, while MMGBSA calculations revealed consistent binding free energies throughout the simulation time. Collectively, the experimental and computational findings provide strong evidence that TAF binds to ct-DNA *via* a minor groove binding mechanism, primarily stabilized by hydrophobic and hydrogen interactions. These results offer deeper insights into the molecular behavior of TAF beyond its known protein targets and may inform future studies on off-target DNA interactions, drug safety profiling, or the design of TAF analogues with enhanced biocompatibility or specificity.

## Conflicts of interest

The authors declare that they have no known competing financial interests or personal relationships that could have appeared to influence the work reported in this paper.

## Data Availability

The datasets generated and/or analyzed during the current study are available from the corresponding author on reasonable request.
